# The isochore patterns of invertebrate genomes

**DOI:** 10.1186/1471-2164-10-538

**Published:** 2009-11-18

**Authors:** Rosalia Cammarano, Maria Costantini, Giorgio Bernardi

**Affiliations:** 1 Stazione Zoologica Anton Dohrn, 80121 Naples, Italy

## Abstract

**Background:**

Previous investigations from our laboratory were largely focused on the genome organization of vertebrates. We showed that these genomes are mosaics of isochores, megabase-size DNA sequences that are fairly homogeneous in base composition yet belong to a small number of families that cover a wide compositional spectrum. A question raised by these results concerned how far back in evolution an isochore organization of the eukaryotic genome arose.

**Results:**

The present investigation deals with the compositional patterns of the invertebrates for which full genome sequences, or at least scaffolds, are available. We found that (i) a mosaic of isochores is the long-range organization of all the genomes that we investigated; (ii) the isochore families from the invertebrate genomes matched the corresponding families of vertebrates in GC levels; (iii) the relative amounts of isochore families were remarkably different for different genomes, except for those from phylogenetically close species, such as the Drosophilids.

**Conclusion:**

This work demonstrates not only that an isochore organization is present in all metazoan genomes analyzed that included Nematodes, Arthropods among Protostomia, Echinoderms and Chordates among Deuterostomia, but also that the isochore families of invertebrates share GC levels with the corresponding families of vertebrates.

## Background

Recent investigations from our laboratory [1] showed that the isochore families of all vertebrate genomes explored are essentially conserved in GC levels (as well as in dinucleotide patterns) and in isochore sizes (with some exceptions in this case). Moreover, at least in eutherian mammals and birds, even the relative amounts of isochore families (*i.e*. the compositional patterns) are largely conserved. It is well established by our previous work that a number of very basic genome properties, such as the distribution of genes and interspersed repeats, DNA methylation, gene expression, replication timing and recombination, are different in GC-poor and GC-rich isochore families (see [2,3], for reviews). These results obviously support the idea of isochores being a "fundamental level of genome organization" [4], at least in vertebrates. We also suggested that the conservation of GC levels of isochore families may underlie chromatin structures [5], and that the conservation of isochore size may be associated with their role in the structure and replication of chromosomes [6].

The above points raise the question as to how general the isochore structure is in the genomes of metazoa. This is a pertinent question because evidence for a compositional heterogeneity was already obtained in our previous work on the genomes of trypanosomes, plasmodium and drosophila [see ref. [2]]

Here we approached this problem by investigating the compositional compartmentalization of genomes from invertebrates in all cases in which either full sequences or at least scaffolds were available. The case of *Apis mellifera *will be presented elsewhere because the presence in this genome of a very GC-poor family (in addition to the other isochore families), which was absent from other insects, required a special analysis. Expectedly, the present analysis broadens our view of the structure and evolution of isochores. In fact, it demonstrates that an isochore organization endowed with a number of shared features is found in the genomes of all metazoans explored in the present work.

## Results

### Isochore patterns

**Figure 1:**
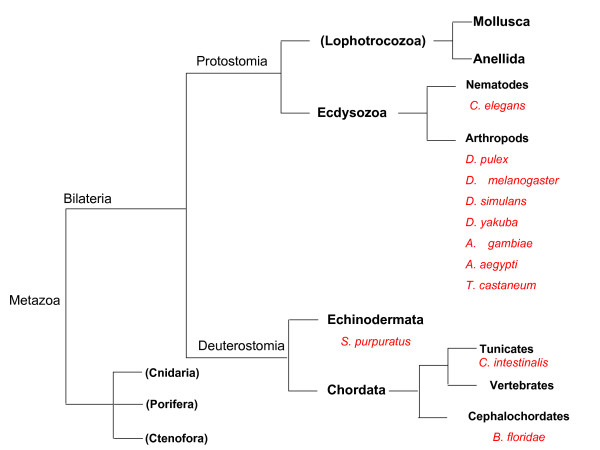
**The location of the species investigated in these genomes are reported in the approximate tree presented**. This is derived from Dunn et al. [7], to which the reader is addressed for the precise tree.

Figure 1, which displays a simplified tree of life (derived from [7]) of metazoans, in order to outline the phylogenetic position of the genomes investigated. Figure 1 is shown to indicate the phylogenetic positions of invertebrate genomes explored. These comprised Nematodes, Arthropods, Echinoderma and Chordata. As shown in Additional File 1, the sizes of the genomes explored covered a 2.5-fold range, 95 to 240 Mb which, added to the 8-fold range of vertebrates, 400 to 3200 Mb, expand the overall range of investigated genome sizes up to a 33-fold value.

**Figure 2:**
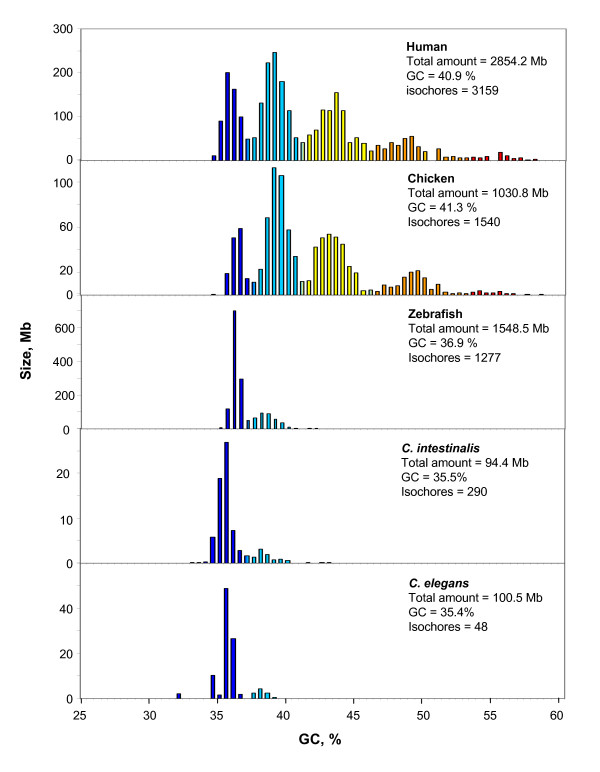
**Distribution of isochores according to GC levels**. The first three histograms, shown for the sake of comparison, display the distribution (by weight) of isochores as pooled in bins of 0.5% GC of some vertebrates: human [9], chicken [11] and zebrafish [10]. The bottom panels show the isochore pattern of *C. intestinalis *and of *C. elegans*. The total amount of DNA calculated from the sums of isochores, GC % and the number of isochores are reported. Colors represent the five isochore families of the human genome. Notice the different scales on the ordinate axis.

**Figure 3:**
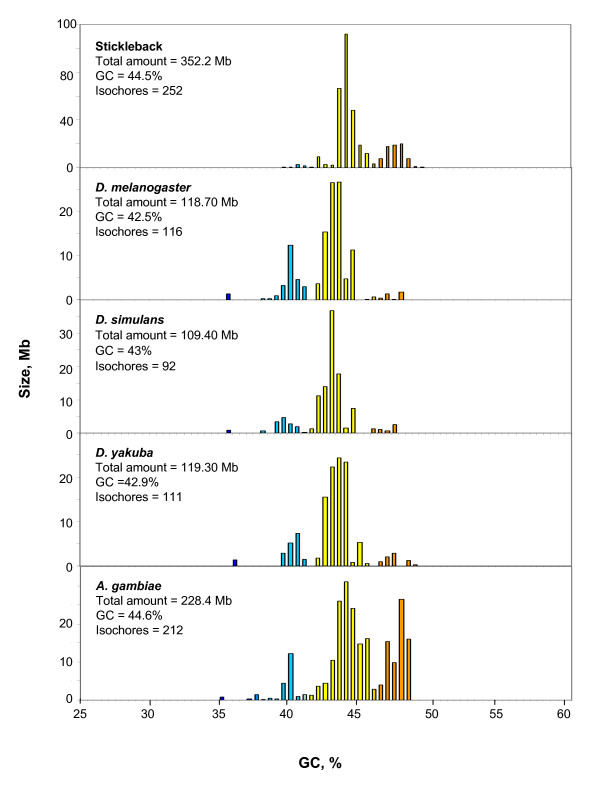
**The distribution of isochores according to GC levels is displayed for *D. melanogaster*, *D. simulans*, *D. yakuba*, *A. gambiae***. The histogram of stickleback is shown for the sake of comparison. For other details see legend of Figure 2.

Table 1A presents the relative amounts of the isochore families of all invertebrates investigated (along with those of human and three fishes that are shown for the sake of comparison). The salient features that appear in the isochore patterns (Figures 2 and 3) will now be outlined. The isochore families of *Ciona intestinalis*, a Urochordate, which is the closest ancestral species to vertebrates [8] are presented in Figure 2 (along with those of three representative vertebrates, human, chicken and zebrafish) in order to show the existence of a major L1 and a minor L2 isochore family. The genome of the nematode *Caenorhabditis elegans *(Figure 2) also displays a very GC-poor genome, consisting essentially of L1 isochores with only a very small amount (less than 10%) of L2 isochores.

**Table 1:** Relative amounts (A), average GC (B) and average size (C) of isochore families from the multicellular eukaryotes under analysis.

	L1	L2	H1	H2	H3	c-value
**A) Relative amount**						
						
***C. intestinalis***	^**(*a*) **^**84.7**	14.9	0.3	---	---	94.4
***C. elegans***	**90.6**	9.4		---	---	100.5
***D. melanogaster***	1.1	20.8	**75.0**	3.1	---	118.7
***D. simulans***	0.8	12.2	**82.0**	0.1	---	109.4
***D. yakuba***	1.1	14.0	**78.7**	6.2	---	119.3
***A. gambiae***	0.5	9.9	**58.3**	31.3	---	228.4
						
***Human***	19.0	37.0	31.0	11.0	3.0	
***D. rerio***	75.7	23.3	---	---	---	1548.5
***O. latipes***	---	71.0	23.7	---	---	725.6
***T. nigroviridis***	---	---	55.3	37.8	---	220.0
						
**B) Average GC**						
						
***C. intestinalis***	35.5	38.3	41.7	---	---	
***C. elegans***	35.4	37.9	---	---	---	
***D. melanogaster***	35.3	40.0	42.9	47.2	---	
***D. simulans***	35.6	39.9	43.1	47.0	---	
***D. yakuba***	35.7	40.1	43.0	47.0	---	
***A. gambiae***	35.8	39.6	44.2	47.2	---	
						
***Human***	36.0	38.9	43.1	48.7	54.5	
***D. rerio***	36.0	38.2	---	---	---	
***O. latipes***		39.9	42.3	---	---	
***T. nigroviridis***	---	---	44.4	48.2	---	
						
**C) Average size (Mb)**						
						
***C. intestinalis***	0.5	0.1	0.06	---	---	
***C. elegans***	^b ^(3.37)	0.5	---	---	---	
***D. melanogaster***	1.3	0.5	(1.6)	0.5	---	
***D. simulans***	0.9	0.4	(2)	0.5	---	
***D. yakuba***	1.3	0.4	(1.7)	0.5	---	
***A. gambiae***	0.6	0.7	(1.27)	1.0	---	
						
***Human***	0.9	0.9	0.8	0.7	0.7	
***D. rerio***	0.8	0.5	---	---	---	
***O. latipes***	---	2.8	0.9	---	---	
***T. nigroviridis***	---	---	0.9	0.7	---	

The c-values in Mb are also reported.

(a)Bold figures indicate the majority isochore families

(b)Figures in parentheses are overestimated (see the text)

Among insects (Figure 3), the three *Drosophila *species studied and *Anopheles gambiae *display three isochore families: a minor L2 family, a predominant H1 family and a H2 family which is barely represented in *Drosophila*, but is rather abundant in *Anopheles*. A very minor (about 1%) L1 component appears to correspond to chromosome 4 in Drosophilids and is barely represented (0.5%) in *A. gambiae*. The isochore families of insects are shown along those of a fish, stickleback.

**Figure 4:**
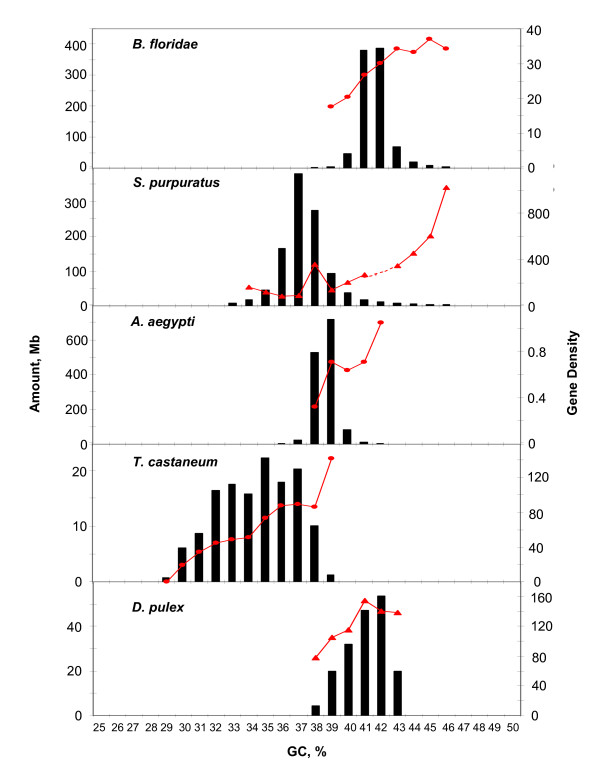
**The scaffolds of *B. floridae*, *S. purpuratus*, *A. aegypti*, *T. castaneum *and *D. pulex *and are pooled in bins of 1% GC**. The gene density (genes/Mb) is calculated on the scaffolds.

The compositional patterns of scaffolds or contigs from *Branchiostoma floridae*, *Strongylocentrotus purpuratus*, *Aedes aegypti*, *Tribolium castaneum *and *Daphnia pulex *are shown in Figure 4. Compositional distribution were generally narrow, covering a range of about 5% GC, with the exception of *T. castaneum *in which case the range was about 10% GC, the center of distribution being lower (33%) than in the other cases.

### GC levels and dinucleotides of isochore families

The average GC levels of the isochore families from the invertebrates investigated are very close to each other and to the corresponding values of vertebrates (see Table 1B).

**Figure 5:**
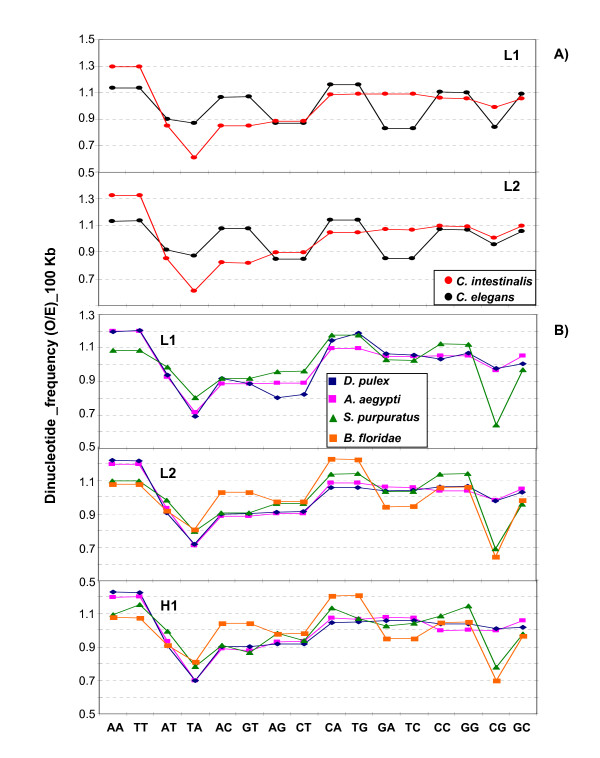
**Observed/expected frequencies A) for dinucleotides in 100-kb DNA segments in the isochore families from *C. intestinalis *and *C. elegans***. and B) for dinucleotides in 100-kb DNA segments in the scaffolds of *B. floridae*, *S. purpuratus*, *A. aegypti*, *T. castaneum *and *D. pulex*.

As far as dinucleotide patterns are concerned, *C. intestinalis *showed a remarkable observed/expected (O/E) pattern in that complementary dinucleotides belonged in two classes, higher and lower than statistical expectation, respectively (see Figure 5). Indeed, AA/TT, AC/GT, CA/TG, CC/GG were in the more frequent class, whereas AG/CT, GA/TC were in the less frequent class. The dinucleotides from all these pairs showed the same frequency. In contrast, TA and CG were less frequent than their complementary dinucleotides AT and GC. Moreover, AT/TA belonged to the less frequent class whereas CG/GC were close to statistical expectation.

Some features of the *C. intestinalis*, a Urochordate, were also found in *C. elegans*, a Nematode. Indeed in *C. elegans *AA/TT, CC/GG and CA/TG were also high, whereas AG/CT were also low, but, in contrast, GA/TC were low and AC/GT were high. Trinucleotides frequencies, showed the expected similarities and differences (see Additional File 2).

**Figure 6:**
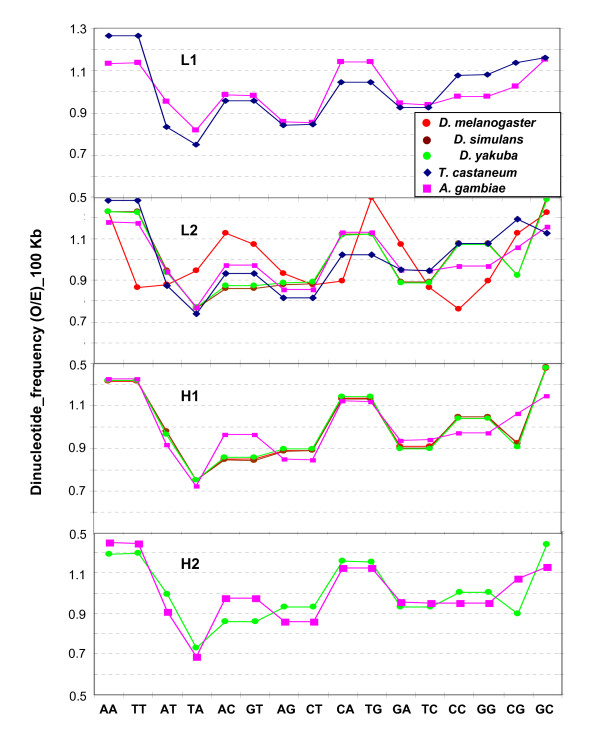
**Observed/expected frequencies for dinucleotides in 100-kb DNA segments isochore families from the Drosophilids, *A. gambiae *and *T. castaneum***.

AA/TT, CC/GC and CA/TG were also high in the insects (and in vertebrates; see ref. [1]; for sake of comparison with human see also Additional File 3), whereas AG/CT was low in insects (but not in vertebrates), and AT/TA were low (TA especially) in all cases (see Figure 6).

Very interestingly, CG was remarkably low in *S. purpuratus *and *B. floridae*. The 0.6 values attained are, however, still much higher than the 0.2 values reached by mammals (see Figure 5B).

### Isochore sizes

The average size from the most represented families of isochores (see Table 1C) is about 0.5 Mb (megabases) for *C. intestinalis*, but higher values were found for *C. elegans *(3.4 Mb) for *Drosophilids *(1.6-2 Mb) and *A. gambiae *(1.3 Mb). The GC-poorest isochore families comprise two size groups, a large one and a small one, as in the case of vertebrates [9-11]. In particular, *C. elegans *showed a number of extremely long GC-poor stretches (see Discussion). In contrast, the GC-richest isochores are characterized by one size group, the small one (see Additional File 4).

### Gene density

**Figure 7:**
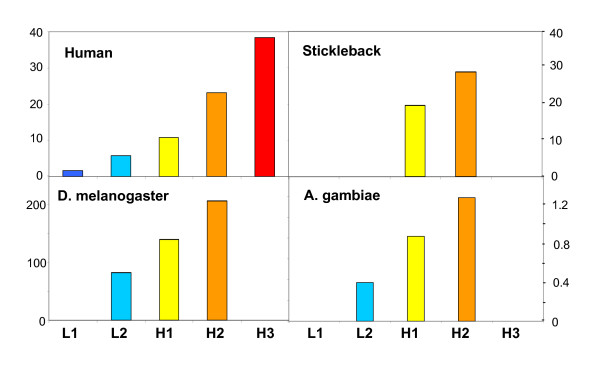
**The histograms represent the gene density in the isochore families**. The gene concentrations of *D. melanogaster *and *A. gambiae *increase with increasing GC in isochore families, as in the case of the genomes from human and stickleback.

Figure 7 displays the gene densities of the isochore families from *D. melanogaster *and *A. gambiae *along with those of two vertebrates, human and stickleback, that are presented for the sake of comparison. In both cases, gene density increased with increasing GC of isochore families. Unfortunately, the data for *C. intestinalis *and *C. elegans *did not permit to establish a reliable ratio between gene densities in the L1 and L2 families because of the small amounts of DNA and number of genes of the minority family. In the case of the genomes only represented by scaffolds, genes were localized on the scaffolds and gene densities were shown to follow the general trend (see Figure 4).

## Discussion

*C. intestinalis *and *C. elegans *essentially show a very predominant L1 isochore family and a minor L2 family, a compositional pattern mimicking that of zebrafish. The case of *C. intestinalis *is of interest because a previous investigation by analytical ultracentrifugation in a CsCl density gradient had shown a remarkable homogeneity at an average molecular weight of 100 kb [12]. The apparent discrepancy can, however, be explained by the fact that the CsCl investigation dealt with random fragments, whereas the present one with 100 kb unique segments. The latter show an average standard deviation of 1.3% GC (Cammarano R., Ph.D. Thesis) a value very slightly above the average standard deviation of 100 kb segments from human isochores from the L1 and L2 families. The dinucleotide patterns (observed/expected) present some significant differences (e.g. TA being much lower than AT in *C. elegans*, but not in *C. intestinalis*), which are also expectedly shown by trinucleotides.

Among insects, the Drosophilids exhibit similar isochore patterns that are intermediate between those of medaka and stickleback with a major H1 family and two minor L2 and H1 families. Another point of interest is the close similarity of GC levels of the isochore families as assessed on Drosophilids and Anopheles (see Table 1B). The compositional pattern of *A. gambiae*, although being mainly represented by H1 isochores, shows a substantial amount of H2 isochores. In fact, the GC-richer isochores of *A. gambiae *have probably been underestimated because of the presence of a large number of 100-kb GC-rich stretches that were pooled with the flanking regions because of the procedure used in assessing isochores (see below).

In the other cases, certainly a number of factors played a role and led to different compositional patterns. Indeed, we already noted that while the large evolutionary changes in isochore patterns occurring between mammals/birds and amphibians/fishes mainly depend upon body temperature, definitely other factors play a role as well in the case of fishes.

The average sizes exhibited by different isochore families of invertebrates showed a greater variability compared to those of the corresponding families from vertebrates [1]. This may be due, however, to artefactual reasons, such as gaps, but also to the experimental approach used. Indeed, in the human genome [9], isochores were taken to be at least 200 kb in size, a condition linked to the need of assessing standard deviations of the 100 Kb segments used in the analysis. Expectedly, this occasionally led to standard deviation higher than 3 % GC within a given isochore. Since however this only concerned ~7% of the human genome such "transition isochores" were accepted.

In the case of *C. elegans *long stretches of DNA very low in GC and belonging to L1 isochores were present and interspersed short L2 isochores were neglected. If one accepts in this case isochores reaching a low size value of 100 Kb, the very long L1 structures are resolved into shorter stretches and the high size values are brought back to the 0.5-1 Mb range. For instance, in the case of *C. elegans *the large size (3.37 Mb) estimated according to the criteria of Costantini et al. [9], is reduced to 1.00 Mb, if 100 Kb segments belonging to the GC range of L2 isochores (and averaging 0.23 Mb in size) are considered separately. This considerations also applies to the large size of major families of insects as indicated by thin 100-kb lines appearing in the GC profiles.

## Conclusion

The major conclusions of the present investigations are the following: (i) an isochore structure appears to be general for all metazoans explored; this raises the question whether, in fact, all eukaryotic genomes are characterized by an isochore structure; current work on plants and unicellular eukaryotes should clarify this point; (ii) the isochore families are generally characterized by GC levels that were identical or very close to those of vertebrates; (iii) differences in dinucleotide patterns (observed/expected values) were found among invertebrates, as well as between invertebrates and vertebrates; in the latter case, the most salient feature was the CpG shortage which is due to the methylation of C in CpG followed by its deamination to T; this feature was also found in *S. purpuratus *and *B. floridae *even if at a lower extent compared to mammals; (iv) the average size of isochore shows a certain variability, which is apparently due at least to a large extent to artefactual reasons, as discussed in the preceding section; (v) no correlation was found between isochore size and genome size in spite of the very large genome size range explored so far; this practical independence of isochore size on genome size stresses their possible correlation with the structure and replication of chromosomes, as suggested by Costantini and Bernardi [6]; (vi) the relative amounts of isochore families are different in different genomes, a situation due in our opinion to the different environmental factors that play a role in determining compositional patterns of genome (for example, if Anopheles has a higher body temperature than Drodophilids it could explain the higher amounts of GC-rich isochores in *A. gambiae*); (vii) gene concentration increases with increasing GC of isochore families, as previously found for vertebrates.

These conclusions are in keeping with some previous suggestions [1]: that (i) the high similarity of GC levels of isochore families may be due to their composition being linked to chromatin structure; (ii) the increasing variability in isochore patterns from warm- to cold-blooded vertebrates and to invertebrates may be correlated with the increasing dependence from environmental factors that affect genome organization and functions; (iii) the distribution of genes seems to be dictated by the need of a certain genomic context, whose composition influences the transcriptional activity, and also the structure and function of the encoded proteins.

## Methods

### Genome and gene sequences

The sequences of the eukaryotic genomes as well as of the genes analyzed in this study were downloaded from different websites (see Additional File 5). Partial, putative, synthetic construct, predicted, not experimental, hypothetical protein, r-RNA, t-RNA, ribosomal and mitochondrial genes were eliminated and then the cleanup program [13] was applied for ridding nucleotide sequence databases of redundancies. For the remaining genes a script implemented by us was used in order to identify the coding sequences beginning with a start codon and ending with a stop codon. The coordinates of genes on the chromosomes were retrieved from the website used for downloading the chromosomes.

### Isochore mapping

The entire chromosomal sequences of the finished genome assembly were partitioned into non-overlapping 100-kb windows, and their GC levels were calculated using the program draw_chromosome_gc.pl [14,15] (http://genomat.img.cas.cz; see Additional File 6).

The methodology used for isochore mapping was described by Costantini et al. [9]. Briefly, isochores are defined by two sets of data, GC levels and their standard deviations. Indeed, when genome sequences are scanned using four window sizes ranging from 12.5 to 100 kb, the final choice of the window is determined by the plateau values reached by the standard deviations [9]. Compositional jumps significantly larger than the standard deviations of isochore GC (1-2%) separate one isochore from the contiguous ones (see Additional File 7 for the coordinates, sizes, GC levels and GC standard deviations of the isochores identified in the analyzed genomes). When isochores are put in bins of 0.5 % or 1% GC, families appear as well defined peaks. In the case of the human genome, five isochore families were found which ranged in GC from 34% to 58% and decreased in relative amounts with increasing GC. These families were also detected by using four different approaches that are based, however, on our boundaries of isochore families [1,16].

In the case of *Branchiostoma floridae*, *Strongylocentrotus purpuratus*, *Aedes aegypti*, *Tribolium castaneum *and *Daphnia pulex*, the genomes have not yet been assembled and only the sequences of scaffolds or contigs (3032 scaffolds, 5477 scaffolds, 4758 supercontigs, 176 contigs, 5192 scaffolds, respectively) are available.

### Nomenclature

As far as the name of each isochore is concerned, we used here, as in previous work, a convention in which the first number in the name represents the chromosome number, the following two letters are the initials of the scientific (latin) name of the Eukaryotes under consideration, and the last number identifies the isochore.

## Abbreviations

Gb: (gigabases); GC: (molar fraction of guanine and cytosine in DNA); kb: (kilobases); Mb: (megabases).

## Competing interests

The authors declare that they have no competing interests.

## Authors' contributions

RC- Downloaded and analyzed the genome and the gene sequences of the Eukaryotes under analysis, performed the analysis on the dinucleotide frequencies and analyzed the data. MC- Conceived the research, identified and mapped the isochores on the analyzed chromosomes and drafted the manuscript. GB- Designed the research and wrote the paper. All contributed to the writing of the manuscript, and all read and approved the final manuscript.

## Supplementary Material

Additional file 1 **Genome sizes**. The figure shows the genome sizes of the invertebrates investigated. Click here for file

Additional file 2 **Observed/expected frequencies for trinucleotides in *C. intestinalis *and *C. elegans***. Observed/expected frequencies for trinucleotides in 100-kb DNA segments in the isochore families from *C. intestinalis *and *C. elegans*. Click here for file

Additional file 3 **Observed/expected frequencies for dinucleotides in human**. Observed/expected frequencies for dinucleotides in 100-kb DNA segments in the human isochore families. Click here for file

Additional file 4 **Isochore sizes**. Size distributions of the isochores in multicellular eukaryotes. A vertical line at 3 Mb is reported as a reference. Click here for file

Additional file 5 **Website and properties for genome and gene sequences**. Genome websites, number of chromosomes, average GC, window sizes at which isochore borders are defined, gene websites and number of genes used to calculate gene density are reported for multicellular eukaryotes. Click here for file

Additional file 6 **Overview of multicellular eukaryotic chromosomes**. Compositional overview of multicellular eukaryotes The color-coded map shows 100 kb moving window plots using the program draw_chromosome_gc.pl [14,15]http://genomat.img.cas.cz. The color code spans the spectrum of GC levels in six steps, indicated by broken horizontal lines, from ultramarine blue (GC-poorest L1 isochores) to red (GC-richest H3 isochores). Click here for file

Additional file 7 **Isochores in invertebrate genomes under analysis**. Coordinates, sizes, GC levels and GC standard deviations of the isochores identified in the invertebrate genomes under analysis. Click here for file

## References

[B1] Costantini M Cammarano R Bernardi G The evolution of isochore patterns in vertebrate genomes BMC Genomics 2009 10 146 10.1186/1471-2164-10-146 19344507 PMC2678159

[B2] Bernardi G Structural and Evolutionary Genomics. Natural Selection in Genome Evolution 2004 Elsevier, Amsterdam, The Netherlands

[B3] Bernardi G The neo-selectionist theory of genome evolution Proc Natl Acad Sci USA 2007 104 8385 8390 10.1073/pnas.0701652104 17494746 PMC1866311

[B4] Eyre-Walker A Hurst LD The evolution of isochores Nat Rev Genet 2001 2 549 555 10.1038/35080577 11433361

[B5] Costantini M Bernardi G The short-sequence designs of isochores from human genome Proc Natl Acad Sci USA 2008 105 13971 13976 10.1073/pnas.0803916105 18780784 PMC2532971

[B6] Costantini M Bernardi G Replication timing, chromosomal bands and isochores Proc Natl Acad Sci USA 2008 105 3433 3432 10.1073/pnas.0710587105 18305168 PMC2265141

[B7] Dunn CW Hejnol A Broad phylogenomic sampling improves resolution of the animal tree of life Nature 2008 452 745 749 10.1038/nature06614 18322464

[B8] Delsuc F Brinkmann H Chourrout D Philippe H Tunicates and not cephalochordates are the closest living relatives of vertebrates Nature 2006 439 965 968 10.1038/nature04336 16495997

[B9] Costantini M Clay O Auletta F Bernardi G An isochore map of human chromosomes Genome Research 2006 16 536 541 10.1101/gr.4910606 16597586 PMC1457033

[B10] Costantini M Clay O Auletta F Bernardi G Isochore and gene distribution in fish genomes Genomics 2007 90 364 371 10.1016/j.ygeno.2007.05.006 17590311

[B11] Costantini M Di Filippo M Auletta F Bernardi G Isochore pattern and gene distribution in the chicken genome Gene 2007 400 9 15 10.1016/j.gene.2007.05.025 17629634

[B12] de Luca di Roseto G Bucciarelli G Bernardi G An analysis of the genome of Ciona intestinalis Gene 2002 295 311 316 10.1016/S0378-1119(02)00734-5 12354666

[B13] Grillo G Attimonelli M Liuni S Pesole G CLEANUP: a fast computer program for removing redundancies from nucleotide sequence databases Comp Appl Biosci 1996 12 1 8 8670613 10.1093/bioinformatics/12.1.1

[B14] Pavlìček A Jabbari K Pačes J Pačes V Hejnar J Bernardi G Similar integration but different stability of Alus and LINEs in the human genome Gene 2001 276 39 45 10.1016/S0378-1119(01)00645-X 11591470

[B15] Pačes J Zika R Pavlìček A Clay O Bernardi G Representing GC variation along eukaryotic chromosomes Gene 2004 333 135 141 10.1016/j.gene.2004.02.041 15177688

[B16] Schmidt T Frishman D Assignment of isochores for all completely sequenced vertebrate genomes using a consensus Genome Biology 2008 9 R 104 10.1186/gb-2008-9-6-r104 PMC2481423 18590563

