# Supplementary figures and images for: The isochore patterns of invertebrate genomes
Source: BMC Genomics. 2009 Nov 18;10:538. doi: 10.1186/1471-2164-10-538 (PMC2783168; doi:10.1186/1471-2164-10-538)

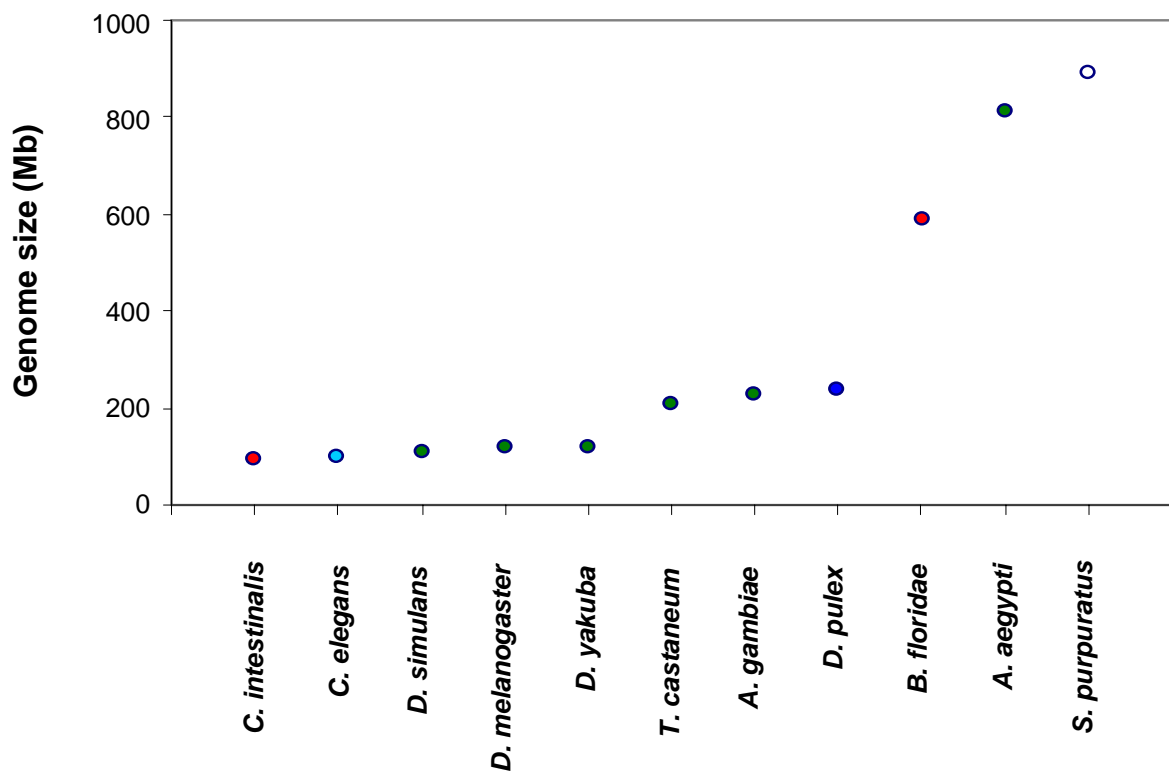

Supplement: Additional file 1 — Genome sizes. The figure shows the genome sizes of the invertebrates investigated. [file 1471-2164-10-538-S1.PDF]

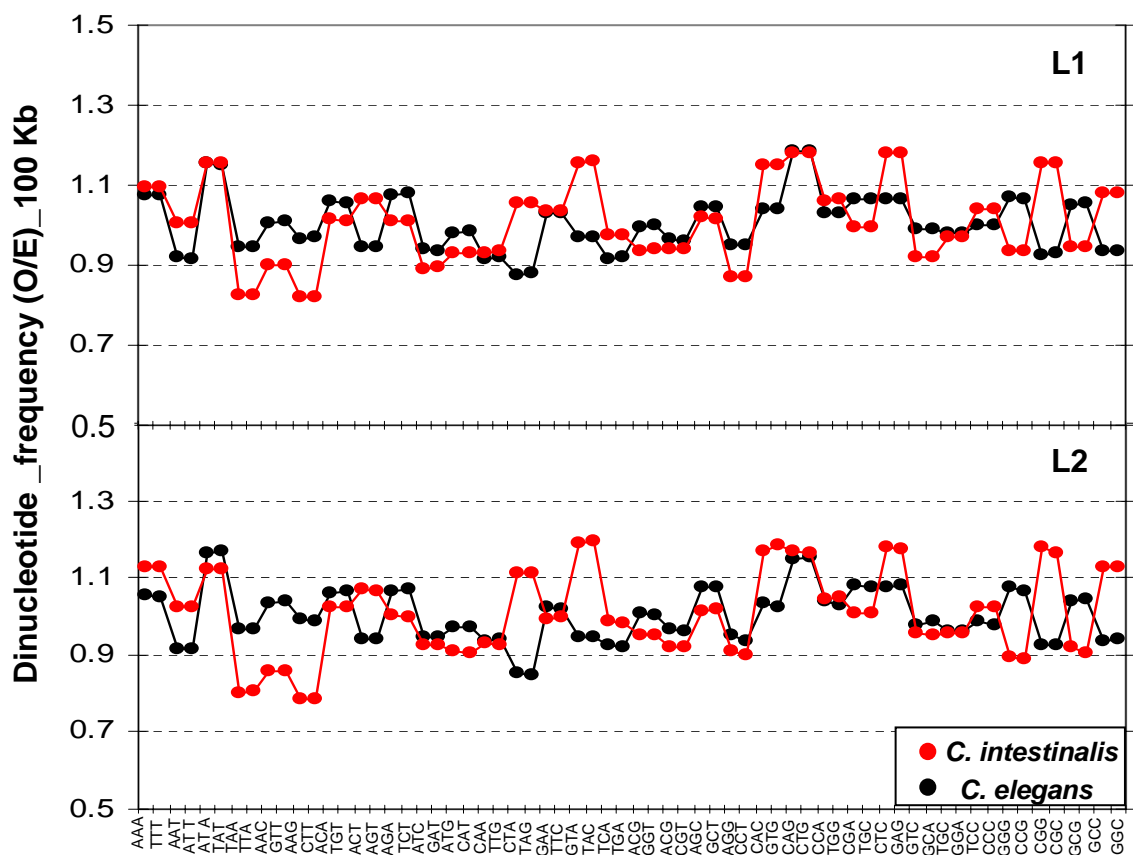

Supplement: Additional file 2 — Observed/expected frequencies for trinucleotides in C. intestinalis and C. elegans. Observed/expected frequencies for trinucleotides in 100-kb DNA segments in the isochore families from C. intestinalis and C. elegans. [file 1471-2164-10-538-S2.PDF]

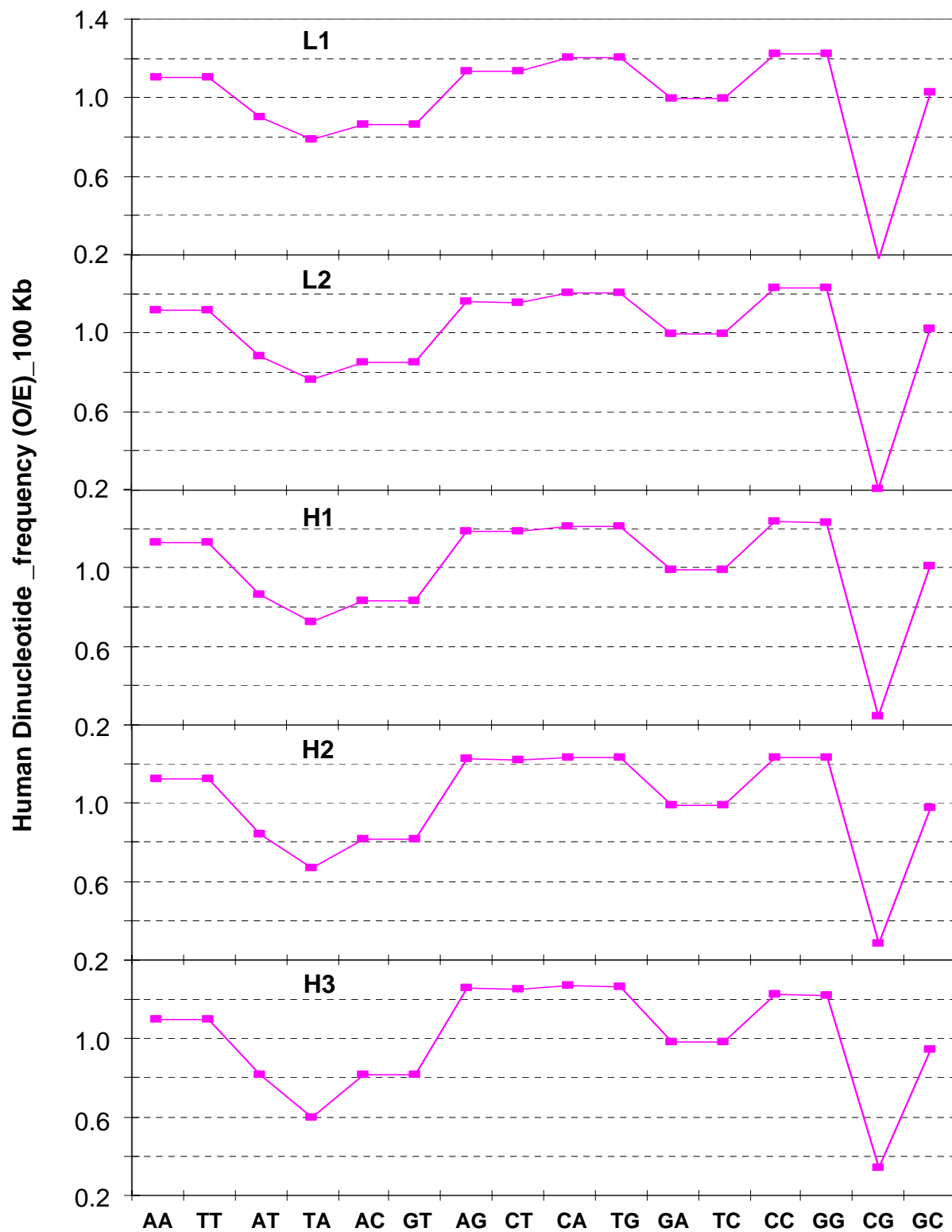

Supplement: Additional file 3 — Observed/expected frequencies for dinucleotides in human. Observed/expected frequencies for dinucleotides in 100-kb DNA segments in the human isochore families. [file 1471-2164-10-538-S3.PDF]

Number of isochores per 0.2 Mb bins

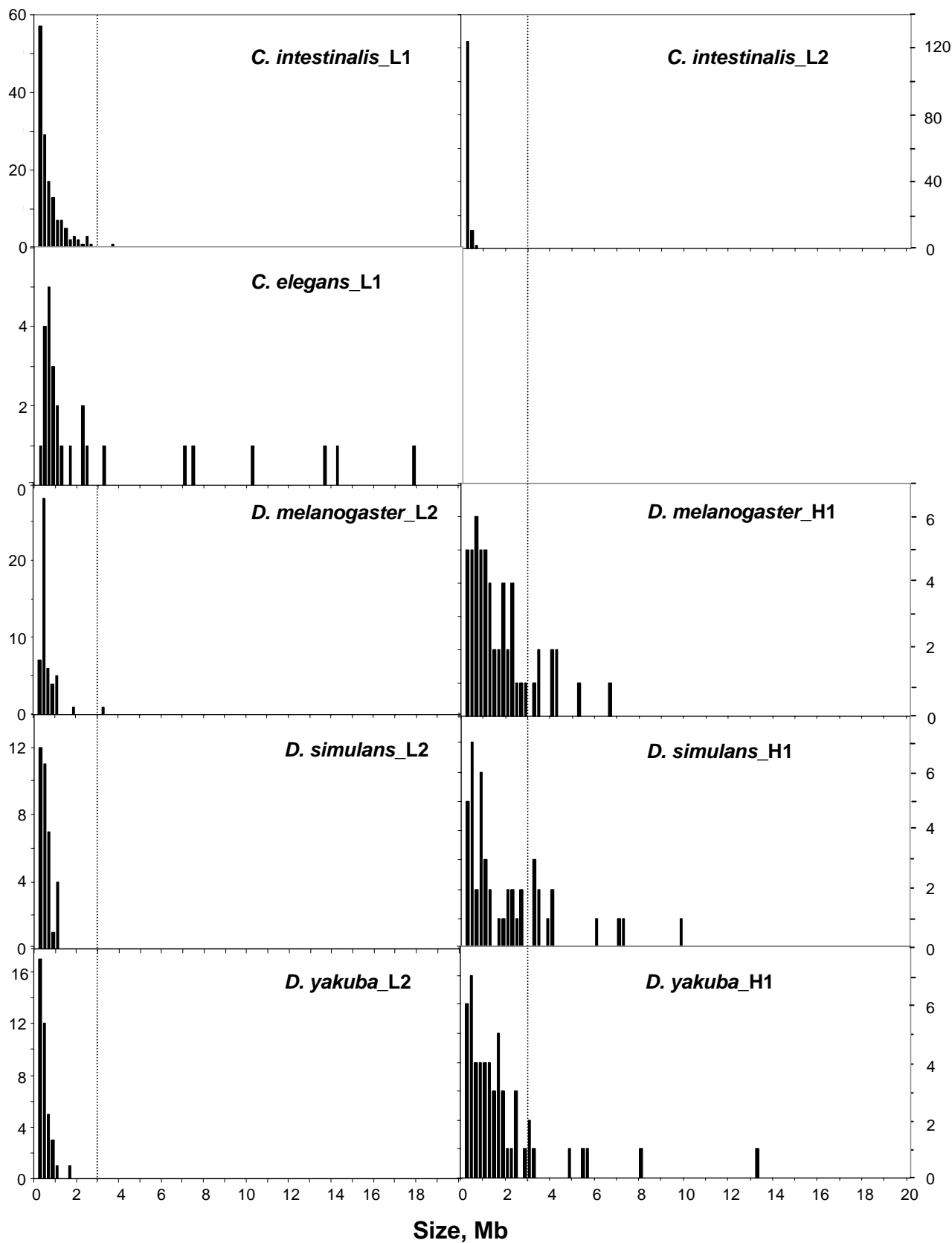

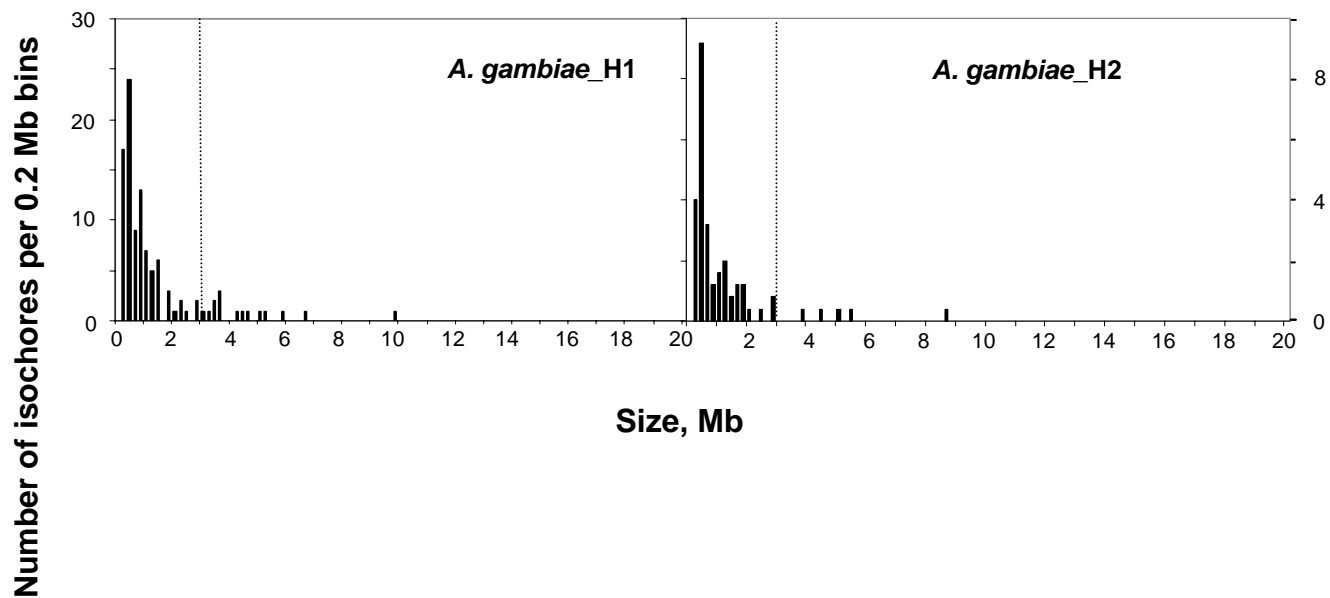

Supplement: Additional file 4 — Isochore sizes. Size distributions of the isochores in multicellular eukaryotes. A vertical line at 3 Mb is reported as a reference. [file 1471-2164-10-538-S4.PDF]

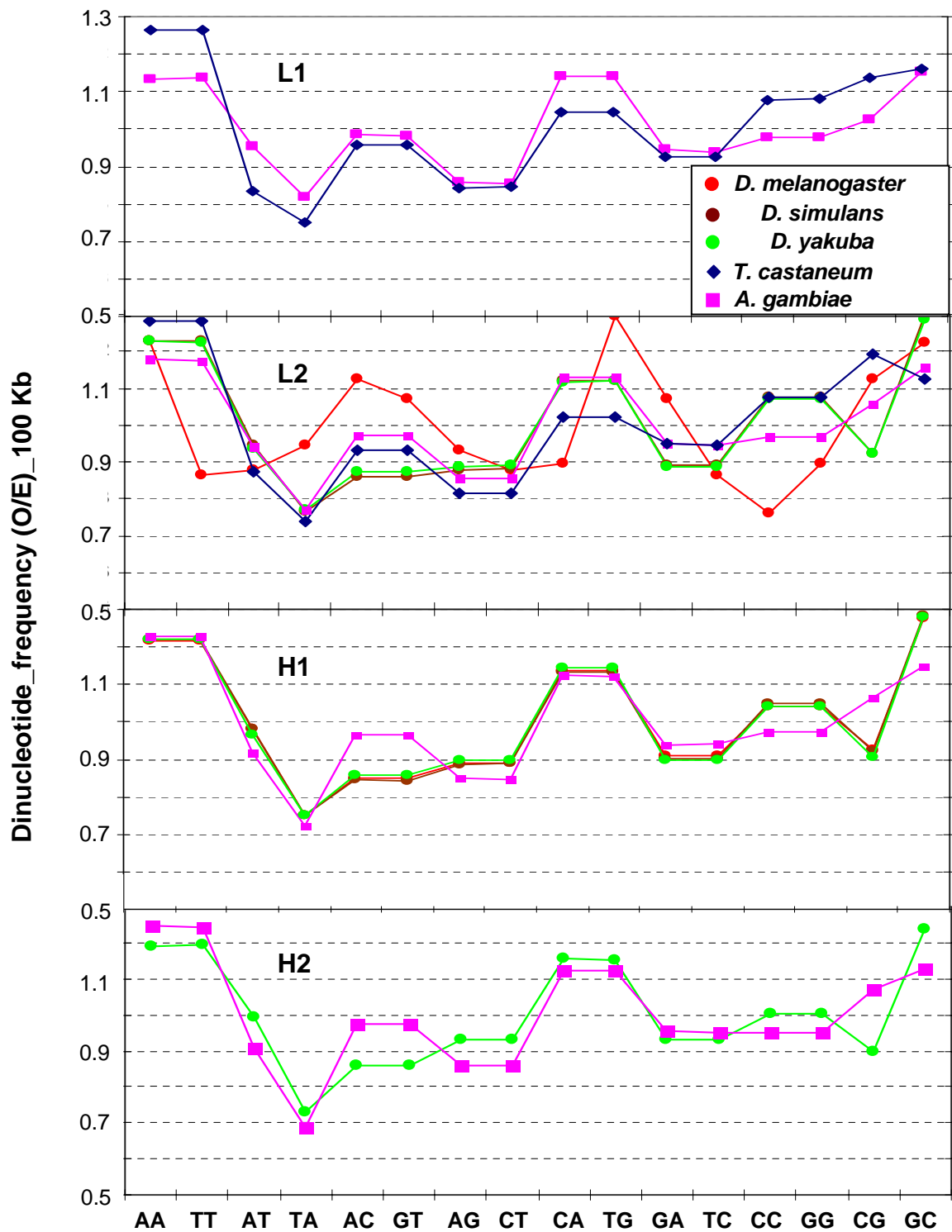

Supplement: Additional file 6 — Overview of multicellular eukaryotic chromosomes. Compositional overview of multicellular eukaryotes The color-coded map shows 100 kb moving window plots using the program draw_chromosome_gc.pl [14,15]http://genomat.img.cas.cz. The color code spans the spectrum of GC levels in six steps, indicated by broken horizontal lines, from ultramarine blue (GC-poorest L1 isochores) to red (GC-richest H3 isochores). [file 1471-2164-10-538-S6.PDF]

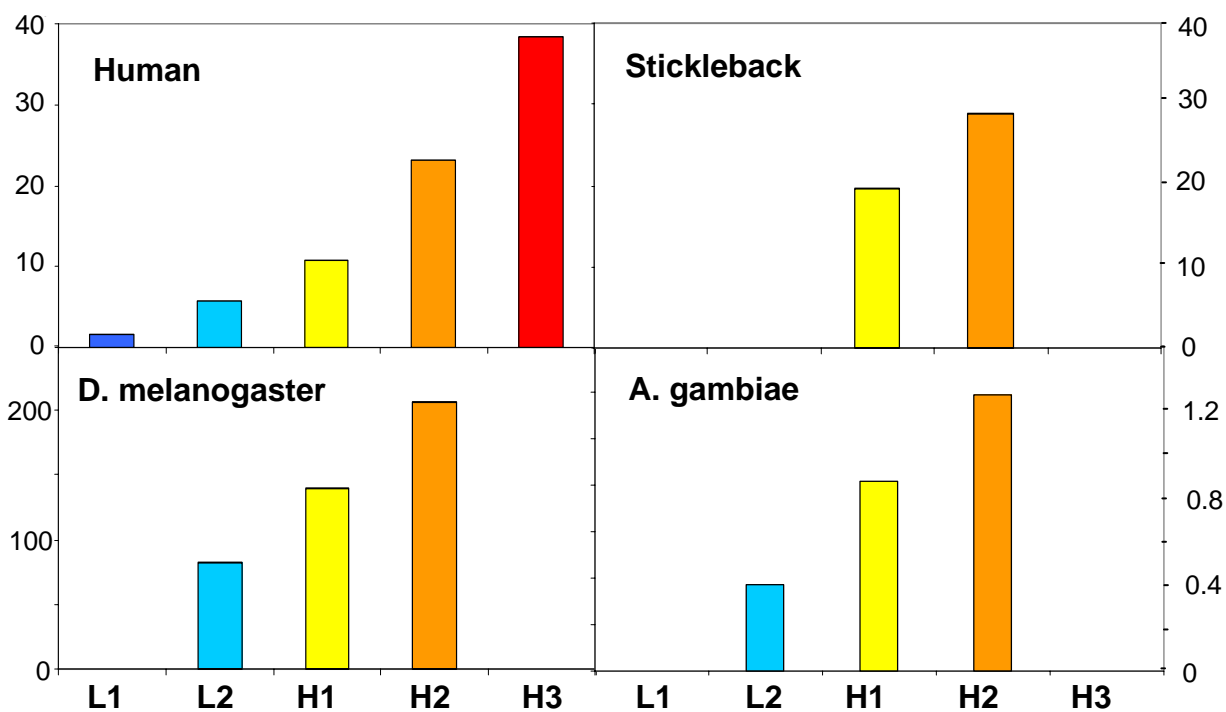

Supplement: Additional file 7 — Isochores in invertebrate genomes under analysis. Coordinates, sizes, GC levels and GC standard deviations of the isochores identified in the invertebrate genomes under analysis. [file 1471-2164-10-538-S7.PDF]
