# Supplementary material for: The isochore patterns of invertebrate genomes
Source: BMC Genomics. 2009 Nov 18;10:538. doi: 10.1186/1471-2164-10-538 (PMC2783168; doi:10.1186/1471-2164-10-538)
Supplement: Additional file 5 — Website and properties for genome and gene sequences. Genome websites, number of chromosomes, average GC, window sizes at which isochore borders are defined, gene websites and number of genes used to calculate gene density are reported for multicellular eukaryotes. [file 1471-2164-10-538-S5.PDF]

Additional File 5. Genome websites, number of chromosomes, average GC, window sizes at which isochore borders are defined, gene websites and number of genes used to calculate gene density are reported for multicellular eukaryotes.

Eukaryotes	Genome_website	Number of chromosomes	GC, %	Window, k	Gene_website	Number of genes
C. intestinalis	JGI; http://jgi.doe.gov/	20	35,6	25	JGI	1778
D. melanogaster	UCSC; http://genome.ucsc.edu/	6	42,5	100	GeneBank	15692
D. yakuba	UCSC; http://genome.ucsc.edu/	6	42,9	100	GeneBank	70
D. simulans	UCSC; http://genome.ucsc.edu/	6	43,0	100	GeneBank	238
A. gambiae	UCSC; http://genome.ucsc.edu/	5	44,6	100	NCBI	6538
T. castaneum	NCBI; http://www.ncbi.nlm.nih.gov/	10	36,4	100		
C. elegans	UCSC; http://genome.ucsc.edu/	6	35,4	100	Ensembl	9763

	Genome_website	Number of scaffolds/contigs	GC, %	Gene_website	Number of genes
B. floridae	JGI; http://genome.jgi-psf.org/Brafl1/Brafl1.home.html	3032	41,4	JGI	25757
S. purpuratus	UCSC; http://genome.ucsc.edu/	5477	37,2	Caltech	15517
A. aegypti	ENSEMBLE; http.ensembl.org/index.html	4758	39,4	GeneBank	315
T. castaneum	NCBI; http://www.ncbi.nlm.nih.gov/ (AAJJ00000000)	176	33,8	NCBI	9165
D. pulex	JGI; http://genome.jgi-psf.org/Dappu1/Dappu1.home.html	5192	41,5	JGI	43258
